# Urgent Arthroplasty Interventions During the COVID-19 Pandemic: Operating Risks in Low-Prevalence Areas

**DOI:** 10.7759/cureus.12197

**Published:** 2020-12-21

**Authors:** Hannah L Burton, Eleanor Burden, Andrew King, Al-Amin Kassam, Matthew J Hubble, Andrew D Toms

**Affiliations:** 1 Trauma and Orthopaedics, Royal Devon and Exeter Hospital, Exeter, GBR; 2 Orthopaedic Surgery, Royal Devon and Exeter Hospital, Exeter, GBR; 3 Orthopaedics, Royal Devon and Exeter NHS Foundation Trust, Exeter, GBR

**Keywords:** covid-19, arthroplasty, elective, orthopaedics

## Abstract

Background and objective

Orthopaedic services have reorganised their delivery of care in response to the severe acute respiratory syndrome coronavirus 2 (SARS-CoV-2) pandemic. In this study, we aimed to share our operating experience during the coronavirus disease 2019 (COVID-19) pandemic and analyse its effect on urgent hip and knee arthroplasty. Our study involved a comparative analysis between a cohort of patients from 2019 (pre-COVID) and another from 2020.

Methods

Tha data relating to patients undergoing urgent operations requiring arthroplasty interventions such as for infection, periprosthetic fracture (PPF) and neck of femur fracture (NOF) between April and July of 2020 and 2019 were reviewed prospectively and retrospectively. Patients were categorised according to the Royal College of Surgeons (RCS) case prioritisation and the COVID-19 risk assessment. Data were collected on 30-day mortality, readmissions, reoperations, complications, length of hospital stay and theatre efficiency. This was analysed, matched and compared. Statistical analysis was performed on categorical variables including the time to the theatre as well as dual consultant operating.

Results

A total of 46 consecutive patients were included in the 2020 cohort with a mean age of 78 years (range: 58-108 years). The median length of stay was 6.5 days (range: 3-35 days) and the median time to theatre for NOF patients was 23.8 hours (range: 16.2-87.7 hours). There were six complications and two deaths; one of the deaths was COVID-19-related. A total of 56 patients were included from 2019 with a mean age of 74.6 years (range: 45-88 years). The median length of stay was five days (range: 1-18 days) and the median time to theatre for NOF patients was 40.8 hours (range: 18.9-167 hours). There were four complications and one death.

Conclusion

Based on our findings, it is safe to perform complex surgery in a region of low community prevalence of COVID-19, and the outcomes were comparable to those from a pre-COVID-19 cohort.

## Introduction

Orthopaedic services, both nationally and internationally, have had to reorganise their delivery of care in response to the coronavirus disease 2019 (COVID-19) pandemic. On the 18th of March 2020, the United Kingdom (UK) government announced that all elective surgical services should be suspended for three months [1]. This was followed shortly by the announcement of a full lockdown on the 23rd of March 2020 [2]. Most hospitals took drastic and immediate actions in an attempt to mitigate the impending surge of COVID-19 admissions. These measures included halting non-urgent elective surgeries immediately, reorganising trauma services to allow for separation from COVID-19 admissions and reducing face-to-face consultations by setting up virtual clinics. Urgent and complex trauma cases continued to be admitted to hospitals, including hip fractures, periprosthetic fractures (PPFs), infected arthroplasties and other general trauma such as open fractures and dislocations. There was a reduced operating capacity as theatre complexes were repurposed for the care of critically unwell COVID-19 admissions, and the remaining space available was shared among all surgical specialties.

We present our experiences of managing complex arthroplasty and PPF fixation during the pandemic at a large teaching hospital in the UK. In the absence of vaccines or effective drug treatments, it remains uncertain as to how long healthcare systems will need to cope with managing services differently. Recently, many organisations have published disease- or specialty-specific guidelines [3,4]; however, few recommendations have suggested resuming elective surgical work and, in particular, performing arthroplasties in this COVID-19 era. As such, we must evaluate and strike a safe balance between the prevention of COVID-19 and the delivery of essential elective surgical care [5]. 

We aim to share our experience of operating during the COVID-19 pandemic and analyse the effect of the pandemic on hip and knee arthroplasty performed during this period through a cohort comparison from the same time period in 2019. This work will help to better inform clinical decision making during and after the COVID-19 pandemic and hopefully aid other centres in restarting elective work safely and appropriately.

## Materials and methods

All patients undergoing procedures related to hip and knee arthroplasty from the 1st April 2020 to 16th June 2020 were included in the 2020 cohort. These cases included primary total hip replacement (THR) for fracture, distal femoral replacement (DFR) for fracture, revision arthroplasty, PPF fixations and debridement or revision procedures for infection [e.g. debridement and implant retention (DAIR) or first stage revision surgery for infection]. Data were collected prospectively using patient notes, electronic records, laboratory reporting systems and picture archiving systems. Patients were categorised according to the Royal College of Surgeons (RCS) guidance for case prioritisation [6] as well as by using the COVID-19 risk assessment [7]. Data were collected on 30-day mortality, readmissions, reoperations, complications, length of hospital stay and theatre efficiency. The same outcome measures were collected retrospectively for a comparative cohort of cases from 1st April to 24th June 2019 and compared against the outcomes mentioned above. These patients were selected from the hospital THR, total knee replacement (TKR) and trauma databases using the procedure codes described above. 

For the 2020 cohort, data regarding COVID-19 screening was collected. Three time intervals were assessed and were defined as follows: preoperative COVID-19 results refer to any swab taken prior to admission or on the day of admission before the patient underwent surgery; admission/postoperative swabs were defined as any tests performed while the patient remained an inpatient and prior to discharge; 30-day post-discharge swabs indicated any COVID-19 swabs performed for whatever reason once the patient had been discharged from the hospital up until 30 days of discharge.

Significance was assessed at the 5% level. Categorical variables such as dual consultant operating were compared with a chi-squared test. Continuous non-normal variables, such as time to theatre for hip fracture [neck of femur fracture (NOF)] patients, were compared using the Mann-Whitney U test. Statistical analysis was performed by one of the authors using Microsoft Excel version 16.39 (Microsoft Corporation, Redmond, WA).

## Results

2020 Cohort

A total of 46 consecutive patients were included in the 2020 cohort (16 males and 30 females), with a mean age of 78 years (range; 58-108 years). Details of the COVID-19 risk status [7] and RCS priority category [6] are provided in Table 1. The median length of stay was 6.5 days (range: 3-35 days) and the median time to theatre for NOF patients was 23.8 hours (range: 16.2-87.7 hours) (Table 2); 44 admissions were due to trauma and two was for elective. A breakdown of operations undertaken is given in Table 3.

**Table 1:** Comparison of the 2019 and 2020 cohorts regarding demographics and potential COVID-19 risk status and RCS prioritisation COVID-19: coronavirus disease 2019; RCS: Royal College of Surgeons

Characteristics	2019 cohort (n=53)	2020 cohort (n=46)
			Age in years (mean)	74.6	78
40-49	1	0
50-59	1	1
60-69	11	5
70-79	20	26
80-89	20	8
90-99	0	5
100-109	0	1
Gender		
Male	20	16
Female	33	30
COVID-19 risk status		
Low	7	3
Medium	15	16
High	20	18
Very high	11	9
RCS priority category		
1a	0	2
1b	18	31
2	2	9
3	16	4
4	17	0

**Table 2:** Representations and readmissions, dual consultant operations, and length of stay ED: emergency department

	2019 cohort (n=53)		2020 cohort (n=46)		
					Representations and readmissions	N	%	N	%	P-value
Representation to ED	3	5.70	2	4.30	0.25
Unplanned readmission	3	5.70	2	4.30	0.25
Dual consultant operation					
	4	7.50	9	17	<0.01
Length of stay (days)	Median	Range	Median	Range	
	5	1-18	6.5	3-35	<0.05
Time to theatre (hours)					
	40.8	18.9-167	23.8	16.2-87.7	<0.05

**Table 3:** Summary of admission types and surgeries undertaken for 2019 and 2020 cohorts ORIF: open reduction and internal fixation

	2019 cohort (n=53)	2020 cohort (n=46)
Admission type		
Trauma	17	44
Elective	36	2
Procedure undertaken		
Revision knee replacement	16	13
Revision hip replacement	21	12
Primary hip replacement/ORIF	15	20
Complex distal femoral replacement	1	1

Complications in 2020 cohort

There were six complications and two deaths in the 2020 cohort; one of the deaths was COVID-19-related, and the other casualty was a centenarian following surgery for a periprosthetic hip fracture. Complications were classified according to the Clavien-Dindo grade [8] and are summarised in Table 4. No patient required a return to theatre in the 30-day follow-up period. Two patients presented to the emergency department (ED), both with symptoms concerning for deep vein thrombosis, but their interval outpatient scans were negative. There were two unplanned readmissions related to complications listed in Table 4.

**Table 4:** Complications by Clavien-Dindo classification *Patient developed deep wound infection following revision THR and died from complication COVID-19: coronavirus disease 2019; EKOS: EkoSonic Endovascular System (Boston Scientific Corporation, Marlborough, MA); THR: total hip replacement

Complications	2019 cohort (n=53)		2020 cohort (n=46)	
						N	%	N	%
Total grade 1	1	1.90%	1	2.20%
Wound seroma	1	1.90%	0	N/A
Wound ooze managed with PICO	0	N/A	1	2.20%
Total grade 2	3	5.70%	4	8.70%
Transfusion	2	3.80%	2	4.30%
Pulmonary embolism managed with anticoagulation	1	1.90%	2	4.30%
Total grade 3	0	N/A	1	2.20%
Pulmonary embolism managed with EKOS®	0	N/A	1	2.20%
Total grade 5	1	1.90%	2	4.30%
Death due to COVID-19-related condition	0	N/A	1	2.20%
Death unrelated to COVID-19	1*	1.90%	1	2.20%
Total complications (all grades)	5	9.40%	8	17.40%

COVID-19 testing

Of note, 89% (41/46) of patients had undergone COVID-19 screening prior to admission or on the day of admission. Of the five patients who did not have pre-admission screening, three had operations before routine testing of inpatients was advised in NHS protocols. One patient tested positive for COVID-19 preoperatively and died of complications from the virus following PPF fixation. No patient tested positive in the 30-day postoperative period. During admission, 12 patients underwent 15 tests postoperatively, and all were negative. Four patients had further COVID-19 testing after discharge but within the 30-day postoperative period, which was all negative (Table 5).

**Table 5:** Summary of the COVID-19 swabs performed on the 2020 cohort of patients throughout the admission and during the 30-day follow-up period COVID-19: coronavirus disease 2019

COVID-19 swab testing	2020 cohort (n=46)
Preoperative COVID-19 swabs (total performed)	41
Positive	1
Negative	40
Admission/postoperative COVID-19 swabs (total performed)	12
Positive	0
Negative	12
30-day post-discharge COVID-19 swabs (total performed)	4
Positive	0
Negative	4

2019 cohort

A total of 53 patients were included in the 2019 cohort (20 males and 33 females), with a mean age of 74.6 years (range: 45-88 years). Details of COVID-19 risk status [7] and RCS priority category [6] are provided in Table 1. The median length of stay was five days (range: 1-18 days) and the median time to theatre for NOF patients was 40.8 hours (range: 18.9-167 hours) (Table 2); 17 admissions were due to trauma and 36 for elective. A breakdown of the operations undertaken is given in Table 3.

Complications in 2019 cohort

There were four complications and one death. The death was related to a deep wound infection following revision THR; other complications are summarised in Table 4. There were three postoperative presentations to ED and three unplanned readmissions.

## Discussion

The COVID-19 pandemic has significantly impacted elective orthopaedic services; since late March 2020, the number of elective orthopaedic operating procedures has fallen by 94% [9]. Trauma operating fell by a smaller margin with a 23.2% reduction in service over the same time period [9]. Operating capacity has been significantly reduced in order to make space for ventilated beds, in line with other hospitals around the country [10]. This has led to specialties sharing operating space in previously ring-fenced orthopaedic theatres. Sharing theatres with other specialties has resulted in concerns about increased arthroplasty infections. Reduced access to theatre space did not result in an increased time to surgery for priority-one patients in this study; this was likely a result of the overall reduction in the number of cases performed. Recent literature has suggested that clinical outcomes must be reviewed and measured to assess the impact on patient outcomes during this period, and that is what we aimed to do in this study [10].

Our data showed that we were able to operate safely and the outcomes in this complex patient group have been comparable to those in a pre-COVID-19 cohort, with similar mortality and morbidity rates between cohorts. Data from Wuhan has indicated that the mortality rate in a similar situation was 20.9% [11]. Wuhan was the centre of the pandemic, and hence a direct comparison is not possible. They were operating without the knowledge of the severity of the virus and with a much higher local reproduction (R) number of at least 3.1 in the early stages of the disease spread [12]. Our region, in contrast, had a low prevalence of COVID-19 during the study period with low numbers of positive cases per 100,000 population [13], and therefore may not be directly comparable to populations such as in the Wuhan study. We have, however, demonstrated the ability to operate safely in this low COVID-19 environment, which can help with the planned restart of elective orthopaedic services.

Our 2020 and 2019 cohorts were similarly matched for age and gender. Patients' COVID-19 risk was comparable, with 39% (18/46) of the 2020 cohort and 38% (20/53) of the 2019 cohort being in the high-risk group, and 20% (9/46) versus 21% (11/53) belonging to the very-high-risk group respectively. The 2020 cohort included cases that required a more urgent range of operations, as classified by the RCS prioritisation guidance, compared to the 2019 cohort. The majority of 2020 operations (33/46) were category 1a or 1b, while the majority of 2019 operations (33/53) were category 3 or 4. This is likely due to the closure of elective work during the 2020 cohort period.

The 2020 cohort included one patient who tested positive for COVID-19 in the preoperative period. Unfortunately, this patient died following major surgery for PPF. This cohort of patients was very vulnerable under any circumstance [14], and COVID-19 increased the risk of morbidity and mortality further. CovidSurg, an international cohort study, has reported a significantly higher risk of dying within 30 days postoperatively if a patient is infected with COVID-19. The risk is reported to be as high as 44.8% in patients over the age of 75 years undergoing major surgery, which would include hip and knee arthroplasty [15]. Morbidity and mortality are likely overestimated in this study as it only included patients formally diagnosed with COVID-19 and may have excluded a significant number of patients who may have contracted the virus but remained in the community and not received formal COVID-19 testing. In addition, they predominantly only reviewed an unwell cohort of patients with no comparison to pre-COVID 19 mortality, as pointed out by a recent BJJ editorial [16]. Other recent literature has found the combination of hip fracture and COVID-19 to be associated with mortality rates as high as 42.9% [17]. For comparison, the 30-day mortality in patients sustaining PPF following hemiarthroplasty has been reported to be 12.5% [18].

In the early stages of the pandemic, the effect of COVID-19 on theatre efficiency, meaning time in theatre, was reduced significantly due to extra care and time taken over airway procedures, donning and doffing of personal protective equipment (PPE) safely and reduced reliance on aerosol-generating equipment in the operating theatre. As these protocols became more familiar, and preoperative testing became the norm, theatre efficiency subsequently improved. Our data for hip fractures for 2020 indicated that time to surgery was shorter than in the pre-COVID-19 era, and this was likely due to the absence of elective operating and the availability of this theatre capacity and staff [9].

Length of stay in the hospital increased during the COVID-19 era for this group of patients according to our data, from a median of five days (range: 1-18 days) to a median of 6.5 days (range: 3-35 days). This was an unexpected finding. We anticipated that the desire to discharge patients from the hospital as early as possible to reduce the risk of COVID-19 would have led to a reduction in length of stay. However, the opposite was observed, but this may be representative of skewed data due to very long stays observed in a minority of patients. It may also represent a theory that the 2020 cohort had significantly more emergency cases than the previous year's cohort, who were largely elective planned cases, and thus had a longer length of stay. It may also be possible that there were fewer care arrangements available from family members and care agencies during the peak of COVID-19 spread. 

A significant change seen during the pandemic was the ease with which complex decisions could be made due to the availability of senior decision-makers from all sub-specialties. This was reflected in the increase in dual consultant operating during the pandemic, which was statistically significantly higher than in our 2019 cohort. This is a model that is being advocated for complex cases by the Getting It Right First Time National Review [19] and one that we are now attempting to maintain as we move into the recovery phase of COVID-19.

The unpredictability in the timeline of the COVID-19 pandemic suggests that it may take many months or years for ‘normal’ elective surgical care to be resumed. Resuming elective procedures should be undertaken in a planned manner so as not to overwhelm the capabilities of healthcare facilities [5]. The current situation in the UK indicates that most regions are experiencing low levels of new infections with the R number estimated to be between 0.7 and 0.9 [20]. A recent study has suggested that the R number fell to as low as 0.57 in May 2020 [21]. In light of this, orthopaedic units will need to restart services in the face of a low but persistent prevalence of the COVID-19. Indeed, the RCS has published guidelines specifically to address such a situation [22]. We believe that this study demonstrates that it is safe, with appropriate precautions and safeguards in place, to recommence arthroplasty work in regions with a low prevalence of COVID-19 taking into account the safeguards that have been put in place. Specific indications and urgency of surgery should be constantly reassessed along with patient-specific risks of COVID-19 complications before proceeding with any intervention [23].

## Conclusions

The COVID-19 pandemic has presented unprecedented challenges to orthopaedic services. Despite the risk from this virus, it is critical that orthopaedic services are resumed as soon as possible to prevent long-term harm and potentially insurmountable waiting lists. Surgical indications and clinical urgency should be continuously reassessed based on local, regional and national situations. This will allow for services to be flexible in a potentially evolving situation and will also ensure that patient care and safety are maintained. This study has demonstrated it is safe to perform complex surgeries in a region with a low community prevalence of COVID-19, and our analysis of the 2020 cohort of patients has shown outcomes comparable to those in a pre-COVID-19 cohort with the best available comparative data.

## References

[1] Iacobucci G (2020). Covid-19: all non-urgent elective surgery is suspended for at least three months in England. BMJ.

[2] (2020). Prime Minister's statement on coronavirus (COVID-19): March 23, 2020. https://www.gov.uk/government/speeches/pm-address-to-the-nation-on-coronavirus-23-march-2020..

[3] Royal College of Obstetrics and Gynaecology, BGCS BGCS (2020). Joint RCOG, BSGE and BGCS guidance for the management of abnormal uterine bleeding in the evolving coronavirus (COVID-19) pandemic. https://www.rcog.org.uk/globalassets/documents/guidelines/2020-05-21-joint-rcog-bsge-bgcs-guidance-for-management-of-abnormal-uterine-bleeding-aub-in-the-evolving-coronavirus-covid-19-pandemic-updated-final-180520.pdf..

[4] (2020). ESC guidance for the diagnosis and management of CV disease during the COVID-19 pandemic. https://www.escardio.org/static-file/Escardio/Education-General/Topic%20pages/Covid-19/ESC%20Guidance%20Document/ESC-Guidance-COVID-19-Pandemic.pdf..

[5] Al-Omar K, Bakkar S, Khasawneh L, Donatini G, Miccoli P (2020). Resuming elective surgery in the time of COVID-19: a safe and comprehensive strategy. Updates Surg.

[6] (2020). Clinical guide to surgical prioritisation during the coronavirus pandemic. https://www.rcseng.ac.uk/coronavirus/surgical-prioritisation-guidance/.

[7] (2020). BOA: re-starting non-urgent trauma and orthopaedic care. https://www.rcsed.ac.uk/media/681197/boa-guidance-full.pdf.

[8] Dindo D, Demartines N, Clavien PA (2004). Classification of surgical complications: a new proposal with evaluation in a cohort of 6336 patients and results of a survey. Ann Surg.

[9] Jenkins P (2020). BOA: the early effect of COVID-19 on trauma and elective orthopaedic surgery. https://www.boa.ac.uk/policy-engagement/journal-of-trauma-orthopaedics/journal-of-trauma-orthopaedics-and-coronavirus/the-early-effect-of-covid-19-on-trauma-and-elect.html.

[10] Al Kaisi K, McClelland D, Dos Remedios I, Belen Carsi M, Malhotra A (2020). BOA: trauma and orthopaedic services during COVID-19 lockdown: The Stoke response to coronavirus pandemic. https://www.boa.ac.uk/policy-engagement/journal-of-trauma-orthopaedics/journal-of-trauma-orthopaedics-and-coronavirus/trauma-and-orthopaedic-services-during-covid-19.html.

[11] Ross GL (2020). Clinical characteristics and outcomes of patients undergoing surgeries during the incubation period of COVID-19 infection. What are the implications for the commencement of elective surgery?. EClinicalMedicine.

[12] Wang H, Wang Z, Dong Y (2020). Phase-adjusted estimation of the number of coronavirus disease 2019 cases in Wuhan, China. Cell Discov.

[13] (2020). Coronavirus (COVID-19) in the UK. https://coronavirus.data.gov.uk/#category=utlas&map=case.

[14] Urban MK (2014). Anesthesia for orthopedic surgery. Miller's Anesthesia.

[15] COVIDSurg Collaborative (2020). Mortality and pulmonary complications in patients undergoing surgery with perioperative SARS-CoV-2 infection: an international cohort study. Lancet.

[16] Ollivere B (2020). “Three meals from chaos”. Bone Jt 360.

[17] Witek A, Sauvé P (2020). BOA: is the combination of a femur fracture a COVID-19 in the over 65s as bad as we think?. https://www.boa.ac.uk/policy-engagement/journal-of-trauma-orthopaedics/journal-of-trauma-orthopaedics-and-coronavirus/is-the-combination-of-a-femur-fracture-a-covid-19.html.

[18] Jennison T, Yarlagadda R (2018). Mortality in patients sustaining a periprosthetic fracture following a hemiarthroplasty. J Orthop.

[19] Briggs T (2020). National review of adult elective orthopaedic services in England: Getting It Right First Time. March.

[20] (2020). The R number and growth rate in the UK. https://www.gov.uk/guidance/the-r-number-in-the-uk#latest-r-number-and-growth-rate.

[21] Riley S, Ainslie KEC, Eales O (2020). Community prevalence of SARS-CoV-2 virus in England during May 2020: REACT study [PREPRINT]. medRxiv.

[22] (2020). England RCoSo. Recovery of surgical services during and after COVID-19. https://www.rcseng.ac.uk/coronavirus/recovery-of-surgical-services/.

[23] Ambrosio L, Vadalà G, Russo F, Papalia R, Denaro V (2020). The role of the orthopaedic surgeon in the COVID-19 era: cautions and perspectives. J Exp Orthop.

